# Recurrent head and neck cancer: United Kingdom National Multidisciplinary Guidelines

**DOI:** 10.1017/S002221511600061X

**Published:** 2016-05

**Authors:** H Mehanna, A Kong, SK Ahmed

**Affiliations:** 1 Institute of Head and Neck Studies and Education, College of Medical and Dental Sciences, University Hospital Birmingham, Heart of England NHS Foundation Trust, UK; 2 Institute of Head and Neck Studies and Education, University of Birmingham, UK; 3 University Hospital Birmingham, UK

## Abstract

This is the official guideline endorsed by the specialty associations involved in the care of head and neck cancer patients in the UK. Recurrent cancers present some of the most challenging management issues in head and neck surgical and oncological practice. This is rendered even more complex by the poor evidence base to support management options, the substantial implications that treatments can have on the function and quality of life, and the difficult decision-making considerations for supportive care alone. This paper provides consensus recommendations on the management of recurrent head and neck cancer.

**Recommendations:**

• Consider baseline and serial scanning with computed tomography and/or magnetic resonance (CT and/or MR) to detect recurrence in high-risk patients. (R)

• Patients with head and neck cancer recurrence being considered for active curative treatment should undergo assessment by positron emission tomography combined with computed tomography (PET–CT) scan. (R)

• Patients with recurrence should be assessed systematically by a team experienced in the range of management options available for recurrence including surgical salvage, re-irradiation, chemotherapy and palliative care. (R)

• Management of patients with laryngeal recurrence should include input from surgeons with experience in transoral surgery and partial laryngectomy for recurrence. (G)

• Expertise in transoral surgery and partial laryngectomy for recurrence should be concentrated to a few surgeons within each multidisciplinary teams. (G)

• Transoral or open partial laryngectomy should be offered as definitive treatment modality for highly-selected patients with recurrent laryngeal cancer. (R)

• Patients with OPC recurrence should have p16 human papilloma virus status assessed. (R)

• Patients with OPC recurrence should be considered for salvage surgical treatment by an experienced team, with reconstructive expertise input. (G)

• Transoral surgery appears to be an effective alternative to open surgery for the management of OPC recurrence in carefully selected patients. (R)

• Consider elective selective neck dissections in patients with recurrent primaries with N0 necks, especially in advanced cases. (R)

• Selective neck dissection (with preservation of nodal levels, especially level V, that are not involved by disease) in patients with nodal (N+) recurrence appears to be as effective as modified or radical neck dissections. (R)

• Use salivary bypass tubes following salvage laryngectomy. (R)

• Use interposition muscle-only pectoralis major or free flap for suture line reinforcement if performing primary closure following salvage laryngectomy. (R)

• Use inlaid pedicled or free flap to close wound if there is tension at the anastomosis following laryngectomy. (R)

• Perform secondary puncture in post chemoradiotherapy laryngectomy patients. (R)

• Triple therapy with platinum, cetuximab and 5-fluorouracil (5-FU) appears to provide the best outcomes for the management of patients with recurrence who have a good performance status and are fit to receive it. If not fit, then combinations of platinum and cetuximab or platinum and 5-FU may be considered. (R)

• Patients with non-resectable recurrent disease should be offered the opportunity to participate in phases I–III clinical trials of new therapeutic agents. (R)

• Chemo re-irradiation appears to improve locoregional control, and may have some benefit for overall survival, at the risk of considerable acute and late toxicity. Benefit must be weighed carefully against risks, and patients must be counselled appropriately. (R)

• Target volumes should be kept tight and elective nodal irradiation should be avoided. (R)

• Best supportive care should be offered routinely as part of the management package of all patients with recurrent cancer even in the case of those who are being treated curatively. (R)

## Introduction

Traditionally patients with recurrence of head and neck cancer (HNC) are considered to have poor prognosis. As a result the majority of these patients are usually treated with palliative intent or receive best supportive care.

Recent systematic review of the literature would suggest, however, that outcomes of the management of recurrence are not as dire as is widely considered. For example, the management of laryngeal recurrence is reported to have good outcomes with rates of up to 71 per cent two-year overall survival.^1^ A recent meta-analysis shows that the outcomes of management of oropharyngeal cancer recurrence appear to have improved significantly over the last two decades, reaching five-year survival of 50 per cent in patients treated surgically.^2^ The latter may be the result of a combination of better patient selection, improved surgical care and the role of the human papilloma virus (HPV) as an aetiological factor.

These improvements in outcomes suggest the need for re-appraisal of the treatment paradigms of HNC recurrence, and the development of specific expertise in the management of recurrence including probably the concentration of expertise in centralised regional or super-regional services.

## Evaluation of the patient with recurrence

Evaluation and careful selection of patients with recurrence is the crux of successful management.^3^ There are several steps in the evaluation process of these patients.

### History

It is important to elucidate the details of the previous treatments that the patient has had, including the chronology and duration since previous treatment. It is also important to identify any toxicity that the patient has experienced from previous treatments as this may have a bearing on any new treatments being offered. The patient's past medical history, and current morbidities and general health state are important, as these will help determine whether the patient is fit enough to receive further curative or palliative active treatments. A smoking and alcohol intake history should be taken. This should especially ascertain whether the patient is currently still smoking or drinking heavily. Finally, a social history of the patient's activities of daily living and their requirements in terms of speech and mobility, as well as their social support structures are important in determining their ability to cope with the demanding treatments that may be required for the management of the recurrence.

### Assessment and staging

*Clinical examination.* Even under anaesthetic, examination can be deceiving if relied upon solely. One study showed a false negative rate of 31 per cent for examination under anaesthetic (EUA) biopsies in 131 patients who showed recurrence within six months of EUA. However, following identification of potential recurrence by scanning, EUA can help provide more information regarding the feasibility of surgical resection and aid planning. Furthermore, a biopsy can be used to assess HPV status, which recently has been found to be of prognostic value in patients with recurrence.^4^ In the longer term, as personalised medicine develops, molecular profiling of the recurrent tumour may provide insights into the most appropriate systemic treatments for that particular tumour.

*Performance status and co-morbidities* Assessment of the patient's overall fitness for anaesthetic and/or systemic therapy is necessary, as that is likely to be an important determinant of whether the patient is able to receive additional treatment in both a curative and a palliative setting.

*Imaging* Positron emission tomography combined with computed tomography (PET–CT) scanning can be extremely helpful in the assessment of recurrence as it can identify the areas of local and nodal recurrence, and importantly distant metastasis. The negative predictive value of PET–CT scan is especially high for recurrence at both the primary site and the neck, approaching values between 93 and 95 per cent and 94 and 100 per cent respectively.^5^ A meta-analysis also showed high sensitivity and specificity for detection of distant metastasis in patients with recurrent HNC (0.92 and 0.95 respectively),^6^ and PET–CT scanning can change the management in 20 per cent of patients with HNC recurrence. In one study, 24 of 123 patients were identified to have silent recurrence or metastasis by PET–CT, of which 50 per cent had thoracic metastasis and 32 per cent had distant metastasis in other sites^7^.

A single CT scan or magnetic resonance imaging (MRI) scan has low accuracy for differentiating between cancer, oedema, and interstitial radiation fibrosis and necrosis. Additional imaging such as an MRI or contrast CT scanning may however be important for planning surgical procedures and outlining radiotherapy (RT).

**Recommendations**

• **Consider baseline and serial scanning with CT or MRI to detect recurrence in high risk patients (R)**
• **Patients with HNC recurrence being considered for active curative treatment should undergo assessment by PET CT scan (R)**

### Decision making for treatment

By combining the findings of the patient assessment process, the following factors need to be considered to help select cases that are appropriate for curative treatment.^3^

• What was the previous disease and what were the treatments given? A review of the extent and features of the previous disease including any poor prognostic features and involved margins is necessary. Furthermore, it is important to elucidate the details of the previous treatment including the levels of neck dissection, the radiotherapy (RT) fields and doses as well as ascertaining any geographic misses and the time since treatment.
• Is there any evidence of distant metastasis? This severely limits the possibility of cure and therefore affects the choice and aggressiveness of treatments to be offered.
• Is it a recurrence at the primary site or a second primary tumour? It is important to ascertain the extent and the size of the recurrence of the primary tumour. Recurrence of a previous tumour has a poorer outcome than a second primary. Furthermore, recurrences in the oropharynx have significantly poorer outcomes than those in the larynx.
• Is there recurrence in the neck? What are the extent and the size of the neck recurrence and is there any evidence of soft tissue extension or extracapsular nodal extension by physical examination and on imaging? The presence of extracapsular extension without the ability to give additional adjuvant treatment significantly reduces the chance of cure and survival.^8^
• Is there evidence of involvement of the carotid arteries, brachial plexus and prevertebral muscles? Involvement of these makes surgical resection unlikely and curative resection almost impossible.
• Can the recurrence be excised surgically with no gross tumour left behind?
• Are there complications and toxicity of previous treatment evident, including osteoradionecrosis or dysphagia? If there are, then the addition of further treatment may result in considerable toxicity and quality of life detriment.
• Is it possible to give RT and/or chemotherapy, taking into account previous treatment, resultant toxicities and time of last treatment?
• What are the potential functional deficits of the proposed treatment for the recurrence?
• What is the state of the patient's reserve, psychological state, general health and family and social support? These factors will be important to consider if the patient is fit and able to undergo further treatment.

## Patient selection criteria

Studies on the outcomes and prognostic factors for the treatment of head and neck recurrence are generally retrospective and of poor quality. They have described, however, several predictors of good outcome which can be classified under three main themes: patient factors, treatment factors and tumour factors.

### Patient factors

The patient factor predictors of good outcome include patients who are non-smokers or who have stopped smoking, have good general heath (ECOG (Eastern Cooperative Oncology Group) status 0–1) and minimal comorbidities,^9^ a good psychological state, good family support, those who are married, and those who are religious or spiritual.

### Tumour factors

Patients with laryngeal recurrence or second primary tumours have better outcomes. Patients with small localised tumours (low T stage (rT1–T2) and a low overall stage^8^ and those with no neck disease on recurrence demonstrate better outcomes. Patients with no nodal extracapsular spread also have better outcomes. Patients who have a recurrence more than 12 months after the end of their treatment appear to do better. Those with recurrence less than six months from treatment completion have persistent disease and a much worse prognosis.^8^ Finally, patients who have HPV positive recurrent disease have longer survival following treatment for recurrence.^4^

### Treatment factors

Patients having surgical resection,^2^^,^^4^ who have received no previous RT or chemotherapy^8^^,^^9^ or have not experienced severe ongoing toxicity from previous treatment appear to have the best outcomes, especially if they have HPV-positive disease.^4^ Patients with resectable disease with no gross tumour remaining after resection and no involved surgical margins^8^ also demonstrate better outcomes, as do patients with no involved vital structures.

## Surgery

### General principles

From the data available, surgery appears to be the modality that is likely to result in the best chance of cure,^2^ especially if there is the possibility of receiving adjuvant treatment post-operatively,^8^ or if the patient has HPV-positive disease.^4^ The aim of surgical treatment is to remove the whole tumour with wide clear margins, leaving no gross residual tumour behind. However, this will usually result in large defects requiring reconstruction. The resulting large functional deficits have to be balanced against the benefit of longer survival and/or or improved palliation.

Surgical salvage is associated with high complication rates and morbidities. The Radiation Therapy Oncology Group (RTOG) 91–11 study reported an overall complication rate of 59 per cent, of which 19 per cent were classified as major complications.^1^ A fistula rate of 30 per cent was reported following salvage laryngectomy after chemoradiotherapy, and 15 per cent if they had been treated with RT. The MD Anderson series of oropharyngeal salvage reported an overall complication rate of 48 per cent.^8^ As a result of that and slower wound healing, patients experience long stays in hospital, which they need to be forewarned about. Such treatment also carries significant costs, which need to be accounted for in reimbursement. Specific interventions that have been shown to reduce complication rates are discussed below.

**Recommendation**

•**Patients with recurrence should be assessed systematically by a team experienced in the range of management options available for recurrence including surgical salvage, re-irradiation, chemotherapy and palliative care (R)**

It is important to note that patients should undergo appropriate and extensive counselling regarding expected survival and functional outcomes, including the long post-operative hospital stays and high complication rates. Early involvement of palliative care physicians in the counselling and treatment of patients, even in situations where curative treatments are being offered, is of benefit to control symptoms and provide psychological support.

### Site-specific factors

#### Larynx

Total laryngectomy is a highly feasible and effective treatment for laryngeal recurrence. In the RTOG 91–11 study, 122 patients recurred after RT or chemoradiotherapy, all of whom had salvage total laryngectomy. The study reported two-year locoregional control rates of 74 per cent and two-year overall survival of 71 per cent.^1^ However, it should be noted that there are several other feasible and highly effective modalities for the treatment of laryngeal recurrence that may also allow preservation of organ function. Transoral laser surgery has been found to be very effective in well-selected patients. In a study of 34 recurrent T1–T4 post-RT failures, 71 per cent were reported to be cured with one or more transoral laser procedure, 29 per cent of patients had tumours that could not be controlled, of which 18 per cent required total laryngectomy and 9 per cent required palliative treatment.^10^ In another study of 53 T1–T4 tumours that recurred after RT,^11^ 42 per cent were cured with one transoral procedure and 16 per cent required more than one procedure, 26 per cent could not be controlled and required total laryngectomy and 11 per cent could not undergo total laryngectomy for recurrence and required palliative treatment. Transoral surgery should however be performed in selected cases by experienced surgeons, as a meta-analysis of transoral laser surgery for radiorecurrent cancers showed around 30 per cent inferior local control compared with open partial laryngectomy.^12^

A systematic review and meta-analysis of 554 patients who underwent salvage open partial laryngectomy concluded that the pooled locoregional control rate was 87.2 per cent (83.3–90 per cent). Pooled overall survival was 83.5 per cent (79.4–87.3 per cent), with a pooled disease-free survival of 91.4 per cent (88.0–94.2 per cent). While 97 per cent of patients underwent successful decannulation, and of the 197 patients where swallowing outcomes were reported, 194 achieved full oral intake.^13^ Supracricoid laryngectomy alone was assessed in a meta-analysis of 103 recurrent T1 and T2 glottic cancer^14^ and local control could be achieved in 85 per cent. In the 15 per cent who had further recurrence, two thirds could be treated further with salvage laryngectomy.

Therefore, total laryngectomy is not the only option for treatment of laryngeal recurrence, and transoral and partial laryngectomy operations are feasible and highly effective. It is recommended that the management of patients with laryngeal recurrence includes input from surgeons who have expertise in transoral and open partial laryngectomy in the recurrence setting, and that this expertise is limited to a small number of surgeons providing regional services.

**Recommendations**

• **Management of patients with laryngeal recurrence should include input from surgeons with experience in transoral surgery and partial laryngectomy for recurrence (G)**
• **Expertise in transoral surgery and partial laryngectomy for recurrence should be concentrated to a few surgeons within the MDT (G)**
• **Transoral or open partial laryngectomy should be offered as definitive treatment modality for appropriate highly-selected patients with recurrent laryngeal cancer (R)**

#### Oropharynx

Recent data suggest that the outcomes of treatment of oropharyngeal recurrence have steadily and markedly improved over the last two decades. In a meta-analysis of five-year outcomes, survival outcomes are reported to have increased from 18 per cent for patients treated before the year 2000 to 51 per cent for patients treated after the year 2000.^2^ It would also appear that the reported complication rates have also decreased considerably over that period of time. This improvement in outcomes may be due to a combination of several factors: better intra- and post-operative care, better use of reconstructive techniques, better patient selection and also the possible role of HPV. Recent data suggest that patients with HPV-positive recurrence of the oropharynx have longer survival rates than patients with HPV-negative recurrence.^4^ Importantly, those patients who are HPV positive and who received surgical resection had significantly better outcomes than the other groups. This would suggest that there is a need for a change in the traditional view that patients with oropharyngeal cancer have very poor outcomes, and therefore are often offered palliative treatments instead of curative resections. It should, however, also be noted that surgical treatment of recurrence carries significant complication rates as well as considerable functional deficits, with reports on return to oral intake varying from 44  to 68 per cent.^8^ Successful resection of oropharyngeal recurrence can be difficult due to the complex three-dimensional anatomy and proximity and adherence to the internal carotid artery. Access procedures through mandibulotomy or lingual release are usually required. Discussion with oncology colleagues regarding areas of highest RT delivery can help plan the siting of the mandibulotomy, as a median mandibulotomy may avoid the areas of the mandible that received the highest RT dose, and therefore avoid the areas at highest risk of osteoradionecrosis. Lingual release is also a good option, but provides limited access to the superior aspects of lateral tonsillar extensions, and may result in higher functional morbidity.

**Recommendations**

• **Patients with oropharyngeal recurrence should have p16 HPV status assessed (R)**
• **Patients with oropharyngeal recurrence should be considered for salvage surgical treatment by an experienced team, with reconstructive expertise input (G)**
• **Transoral surgery appears to be an effective alternative to open surgery for the management of oropharyngeal recurrence in carefully selected patients (R)**

Recently the advent of transoral surgery, and especially transoral robotic surgery (TORS), has facilitated better transoral access to the oropharynx.^15^ This approach is now being utilised for surgical resection of smaller OPC recurrences with good outcomes. A recent multicentre case–control study showed that salvage patients treated with transoral robotic surgery had significantly lower incidence of tracheostomy, feeding tube use, and shorter hospital stay, with significantly decreased incidence of positive margins and significantly higher survival than matched patients treated with open surgery.

#### Nasopharynx

This is the one area traditionally where re-irradiation has been employed for salvage treatment, particularly where the recurrent disease is limited to the confines of the nasopharynx without extensive invasion of the bone of the skull base or intracranial structures. In areas of the world where major centres treat large numbers of these patients, notably Southern China, Hong Kong and Singapore, surgery for localised recurrent disease has been undertaken by means of maxillary swing or other forms of anterior mid-facial approaches. With varying degrees of nasopharyngectomy, cure rates in selected patients have been reported in the region of 40 per cent at five years.

#### Sinus and nasal cavity

Despite the rarity of these tumours and the diversity of pathology in these areas, salvage treatment can achieve good long-term cure rates in carefully selected patients. Endoscopic endonasal surgery is showing comparable outcomes and is the treatment of choice in certain situations for both primary and recurrent disease when compared with conventional open approaches.

Many recurrent tumours such as adenoid cystic carcinoma, chondrosarcoma, intestinal type adenocarcinoma and olfactory neuroblastoma will need a multimodality, multidisciplinary approach, which can only be effectively provided in large centres that have the expertise both in endonasal and in open anterior and anterolateral craniofacial resection. The tumour biology as well as its location determines the best approach. Oncological goals do not change in the endoscopic endonasal route with the goal being negative resection margins. En-bloc resection is often not possible. Despite this, outcomes in both overall survival and disease-free survival are comparable with open approaches and should be considered as a viable treatment option for recurrences.

#### Neck and nodal disease

Neck dissection in the salvage context may carry higher complication rates than in the primary setting. The type of neck dissection also has a bearing on complication rates, with modified radical neck dissections or radical neck dissections carrying higher major complication rates than selective neck dissections in the salvage setting. Furthermore, neck dissection was found to be a significant risk factor for pharyngocutaneous fistula after laryngectomy in a meta-analysis.^16^ Studies looking at avoiding neck dissection in patients with recurrence at the primary site with no clinical evidence of nodal metastasis have shown that whilst the neck dissection is associated with higher complication rates, there was also a lower regional failure rate. On the other hand, other studies have found the pre-operative clinical staging of nodal status in patients undergoing salvage laryngectomy to be highly accurate.^17^ Therefore it would appear that undertaking elective neck dissections in patients with N0 necks following recurrence should be considered, especially in patients with advanced recurrences.

As for patients with proven nodal recurrent disease, selective neck dissection is also as effective as modified radical neck dissections, but potentially carries less morbidity.^18^ The evidence would suggest that using selective neck dissection reduces complication rates and results in similar control rates to more radical neck dissection in recurrence patients who have N0. Indeed, some have suggested that superselective neck dissection is also effective, although the evidence level for this is weak.^19^

**Recommendations**

• **Consider elective selective neck dissections in patients with recurrent primaries with N0 necks, especially in advanced cases (R)**
• **Selective neck dissection (with preservation of nodal levels, especially level V, that are not involved by disease) in patients with nodal (N+) recurrence appears to be as effective as modified or radical neck dissections (R)**

### Reducing complications in salvage surgery

There are interventions that are proven to reduce complications in salvage surgery. These include the following:

• Use of Montgomery salivary bypass tubes has been shown to decrease fistula rates and has also been shown to be cost-effective in laryngectomy^20^
• The use of flap closure for pharyngeal defects if there is any tension on wound closure has been shown to decrease fistula rates. A meta-analysis showed that use of a vascularised flap to augment the circumference or support the repair reduces the risk of fistula formation by one-third.^13^ Flap reconstruction also reduces stricture rates and tube dependence compared with primary closure. The use of a pectoralis major pedicled-flap or a free flap is therefore recommended
• In patients where there is no tension at the anastomotic site, interposition flap reinforcement of the suture line has been shown to decrease fistula rates. This may be undertaken using a pectoralis major myofascial pedicled flap or an interposition free flap, both of which have been shown to reduce fistula rates^13^
• Secondary puncture has also been shown to reduce fistula rates in post-chemoradiotherapy salvage laryngectomies. Although no literature evidence exists, avoidance of three-point junctions in skin incision through the use of horizontal incisions (e.g. Attee or MacFee) may help reduce wound breakdown.

## Palliative chemotherapy

Patients receiving only palliative care have an average overall survival of four months after diagnosis. Outcomes from studies of palliative chemotherapy generally show longer survival rates, depending on the regimen. However, no large well-designed randomised trial has been undertaken to definitively show an overall survival benefit of palliative chemotherapy over the best supportive care in these patients. Several chemotherapy regimens, either single agent or combination treatments have been tried in recurrent head and neck squamous cell carcinoma patients with different results. The active single agents in head and neck squamous cell carcinoma patients with response rates greater than 15 per cent include methotrexate, bleomyin, cisplatin, carboplatin, paclitaxel, docetaxel, cyclophosphamide, doxorubicin, hydroxyurea, vinblastine and fluorouracil (5-FU). Various randomised trials have been undertaken to compare different chemotherapy regimens in recurrence patients. Combination treatment has shown higher response rates than the single-agent therapy.

In comparison with PF (cisplatin 100 mg/m^2^ and 5-FU 750 mg/m^2^ days 1–5 every three weeks) in a randomised controlled trial, TPF induction chemotherapy (docetaxel 75 mg/m^2^, cisplatin 75 mg/m^2^ and 5-FU 750 mg/m^2^ days 1–5 every three weeks) was shown to yield a higher objective response rate as well as increased median progression-free and overall survivals in unresectable head and neck cancer patients without distant metastasis. However, this regimen is mainly used as induction chemotherapy before radical treatment for curative patients and it is not normally used as first line treatment in recurrent or metastatic head and neck squamous cell carcinoma patients with unresectable disease due to significant toxicities associated with this regimen.^21^ Some of the selected chemotherapy regimens commonly used in palliative head and neck squamous cell carcinoma patients are listed in Table I.

**Table I:** Selected palliative chemotherapy regimens commonly used in recurrent or metastatic head and neck squamous cell carcinoma patients (modified from references 1–2)

Regimens	Usual doses	Response rate (%)	Reference
Cetuximab/5-FU/cisplatin	Cisplatin IV 100 mg/m^2^ q3w 5FU IV 1000 mg/m^2^ d1–4 q3w cetuximab IV 400 mg/m^2^ loading dose and 250 mg/m^2^ maintenance dose q1w	36	Gao *et al*.^6^
Cisplatin/5-FU	Cisplatin IV 100 mg/m^2^ q3w 5FU IV 1000 mg/m^2^ d1–4 q3w	27–50	Jayaram *et al*.^2^, Zafereo *et al*.^8^, Paleri and Kelly^9^
Cisplatin/paclitaxel	Cisplatin IV 75 mg/m^2^ q3w paclitaxel IV 175 mg/m^2^ q3w	26–41	Zafereo *et al*.^8^, Steiner *et al*.^10^
Cisplatin/docetaxel	Cisplatin IV 75 mg/m^2^ q3w docetaxel IV 75 mg/m^2^ q3w	53	Roedel *et al*.^11^, Ramakrishnan *et al*.^12^
Carboplatin/paclitaxel	Carboplatin IV AUC 6 q3w with paclitaxel IV 200 mg/ m^2^ q3w or carboplatin IV AUC 2 q1w with paclitaxel 80 mg/m^2^ q1w	27 52	Paleri *et al*.^13^, Marioni *et al*.^14^
Docetaxel	Docetaxel 75–100 mg/m^2^ q3w	21–42	White *et al*.^15^, Paydarfar *et al*.^16^
Paclitaxel	Paclitaxel 80–100 mg/m^2^ q1w paclitaxel 175 mg/m^2^ q3w	13–40	Pezier *et al*.^17^, van der Putten *et al*.^18^
Methotrexate	Methotrexate 40–60 mg/m^2^, q1w	10–77	Dunsky *et al*.^7^, Robbins *et al*.^19^, Murray *et al*.^20^

IV =  intravenous; q3w = every three weeks; q1w = every week; d1–4 = days 1–4

*Note*: some of the trials used different doses and regimens than those listed as ‘usual’ doses.

**Recommendations**

• **Use salivary bypass tubes following salvage laryngectomy (R)**
• **Use interposition muscle-only pectoralis major or free flap for suture line reinforcement if performing primary closure following salvage laryngectomy (R)**
• **Use inlaid pedicled or free flap to close wound if there is tension at the anastomosis following laryngectomy (R)**
• **Perform secondary puncture in post CRT laryngectomy patients (R)**

Since a majority of head and neck squamous cell carcinoma tumours express or overexpress epidermal growth factor receptor, the epidermal growth factor receptor inhibitors including cetuximab has been tried in these patients. A phase III randomised trial of cisplatin plus placebo compared with cisplatin plus cetuximab in metastatic and/or recurrent head and neck cancer was done and it was shown that addition of cetuximab to cisplatin significantly improved response rate but did not significantly improve progression-free and overall survival.^22^ The addition of cetuximab to platinum-based chemotherapy (either cisplatin 100 mg/m^2^ or carboplatin are under the curve 5 with 5-FU 750 mg/m^2^ days 1–4 every three weeks) improved objective response rate, median progression-free and overall survivals compared to platinum-base chemotherapy alone (EXTREME trial).^23^ This regimen is recommended as the first-line systemic treatment for recurrent and metastatic head and neck squamous cell carcinoma patients with good performance status in many centres. However, the choice of EXTREME regimen as first-line treatment will depend on individual patient circumstances and performance status. In England, cetuximab in addition to 5-FU and platinum chemotherapy could be prescribed in the NHS through the cancer drug fund although this fund is not available in other parts of the UK and may only be available in the a short term. As the regimen is associated with high frequencies of toxicities, not all patients can tolerate or complete the treatment.

For patients who are deemed to be unfit to have EXTREME regimen, a modified version of cetuximab and a platin or reduced doses have been used for some patients. In addition, if cetuximab is not used or not available, many centres will use the combination platinum-based regimens (without cetuximab) as first-line treatment for these recurrent head and neck squamous cell carcinoma patients, including those regimens listed in Table I.

Once patients have progressed on platinum based chemotherapy, the prognosis is extremely poor and there is no standard second-line or third-line therapy for these patients. In some cases, another platinum-based combination chemotherapy can be given as second line, e.g. carboplatin and paclitaxel. However, some of these patients may have deteriorating or poor performance status and further combination chemotherapy treatment may be poorly tolerated. In addition, some patients may be platinum-resistant and are unlikely to benefit from further platinum-based chemotherapy. For second- or third-line chemotherapy, single agent taxane (paclitaxel or docetaxel) or methotrexate has also been used in patients who still have relatively good performance status.

For patients who are unfit to have palliative chemotherapy, best supportive care may be the best option, since palliative chemotherapy may worsen their quality of life without a survival benefit. This decision needs to be made by the doctors and patients together, with the involvement of a palliative care physician, focusing on the benefits of palliative chemotherapy *vs* the risks of treatment toxicity.

Patients with non-resectable recurrences being considered for palliative treatment should be offered the opportunity to participate in clinical trials of new therapeutic agents, including immunotherapy. If such trials are not available locally, patients should be referred to centres that offer these trials.

**Recommendations**

• **Triple therapy with platinum, cetuximab and 5-fluorouracil appears to provide the best outcomes for the management of patients with recurrence who have a good performance status and are fit to receive it.  If not fit, then combinations of platinum and cetuximab or platinum and 5-FU may be considered (R)**
• **Patients with non-resectable recurrent disease should be offered the opportunity to participate in Phase I-III clinical trials of new therapeutic agents (R)**

## Re-irradiation

Most patients with recurrence will have had previous radical RT, which would have reached the maximal acceptable tolerance dose for critical organs such as spinal cords and/or brainstem. Therefore, re-irradiation of these patients carries significant potential risks and complications.

### Patient selection

Data on patient selection for chemo re-irradiation is sparse, with comorbidity and pre-existing organ dysfunction being the most important prognostic factors for patients undergoing re-irradiation. Other prognostic factors include interval from previous radiation, recurrent tumour stage, tumour bulk at re-irradiation, and re-irradiation dose.^24^

### Re-irradiation using conventional and older RT techniques for unresectable recurrent cancers

Some single centre and phase 2 studies have shown very good control rates for re-irradiation of recurrent tumours with prolonged survival rates. However, replication of these results in phase 3 studies has not materialised, probably reflecting in part the importance of specialist expertise and careful patient selection. At the Gustave-Roussy Institute, full-dose re-irradiation was given to 169 patients with unresectable head and neck cancer, in the form of either RT alone or with concurrent chemotherapy (5-FU and hydroxyurea or mitomycin, 5-FU and cisplatin). The overall survival (OS) rate was 21 per cent at 2 years and 9 per cent at 5 years, with a median survival time of 10 months for the whole population. In the RTOG 96–10 study, 86 patients received re-irradiation with 5-FU and hydroxyurea. The two- and five-year survival rates were 15.2 and 3.8 per cent respectively with overall grade 3–4 acute toxicities of 56 per cent, grade 3–4 late toxicities of 22 per cent and deaths in 8 per cent of patients.^25^ In the RTOG 99–11 study, recurrent head and neck cancer patients received twice-daily radiation (1.5 Gy per fraction bid 5 days every 2 weeks with low-dose paclitaxel and cisplatin). The estimated one- and two-year OS rates were 50.2 and 25.9 per cent, respectively. The study also showed 28 per cent grade 4–5 acute toxicities and 11 per cent treatment-related deaths.

A randomised phase III trial (Groupe d'Oncologie Radiotherapie Tete Et Cou (GORTEC) 98–03) compared re-irradiation with 5-FU and hydroxyurea chemotherapy with palliative methotrexate monotherapy in patients with recurrent or a second primary head and neck squamous cell carcinoma.^26^ Despite the promising phase II studies, this phase III study showed that re-irradiation with concurrent chemotherapy did not improve OS compared with methotrexate alone (23 per cent *vs* 22 per cent at one year, NS). There were however four complete responses in the re-irradiation arm, and none in the chemotherapy alone arm. Twenty-eight per cent had grade 3 late toxicity in the re-irradiation arm compared with 9 per cent in the chemotherapy arm. The trial was closed prematurely and thus no definite conclusion could be drawn.

The Groupe d'Étude des Tumeurs de la Tête et du Cou (GETTEC) and Groupe d'Oncologie Radiotherapie Tête Et Cou (GORTEC) undertook a randomised study examining the efficacy of adjuvant chemo re-irradiation after salvage surgery. The study included patients who had salvage surgery with no gross residual disease and a good performance status. Patients were randomised to either observation or post-operative chemo re-irradiation (FHX (5-fluoro-uracil, hydroxyurea and radiation) regimen, daily radiation to 60 Gy). Patients in the post-operative chemo re-irradiation arm had significantly improved locoregional control (49 per cent *vs* 25 per cent) and disease-free survival. However, there was no significant difference in overall survival due to an increase in treatment-related deaths and second primary tumours following chemo re-irradiation, with 40 per cent of patients experiencing grade 3 or 4 late toxicity in the chemo re-irradiation arm, compared to 10 per cent in the observation arm.

### Re-irradiation with intensity-modulated radiotherapy (IMRT)

Intensity-modulated radiotherapy (IMRT) can potentially limit the dose to critical areas. At the same time, however, it may increase the dose to surrounding non-critical areas. Therefore, it is not yet completely clear what the balance of benefit and harm will be. In one study, 105 patients with recurrent head and neck cancer underwent re-irradiation using IMRT (75 of whom also received concurrent chemotherapy) and the two-year locoregional progression-free survival and overall survival rates were 42 and 37 per cent, respectively. The acute and late grade 3 toxicities were reported in 23 and 15 per cent of patients respectively. In another study, 84 patients underwent re-irradiation using IMRT (20 per cent received concurrent chemotherapy), five-year locoregional control and overall survival were 40 and 20 per cent respectively, with grade 3 acute and late toxicities of 31 and 13 per cent. Although there was no grade 5 acute toxicity, there were two fatal vascular ruptures during follow-up.

### Re-irradiation with biological therapies

The combination of an epidermal growth factor receptor inhibitor, cetuximab, with RT has been shown to significantly improve overall survival at five years compared with RT alone for locoregionally advanced head and neck cancer. Therefore, there is also rationale for combining cetuximab with re-irradiation in recurrent head and neck cancer patients. One recent study showed a median overall survival of 10 months in recurrent head and neck cancer patients retreated with stereotactic body radiation therapy plus cetuximab. Acute and late grade 3 toxicity was observed in 6 per cent of patients, which seems to be much lower than that of re-irradiation and chemotherapy.

### Toxicity of chemo re-irradiation

Chemo re-irradiation carries risk of very severe life-threatening toxicity, which has to be weighed against the relative survival benefit, and quality of life detriment. The resultant acute major toxicities are similar to those of primary chemoradiotherapy, including mucositis, dermatitis and hematologic suppression. These toxicities generally resolve after the completion of therapy, and most patients recover with supportive measures, although treatment interruptions may be necessary. Compared with re-irradiation alone, the addition of concurrent chemotherapy significantly increases acute toxicities.

Late toxicities are generally less predictable and irreversible, and therefore carry a higher potential for problems. In RTOG 9610, the cumulative incidence of grade 3+ late toxicity in patients surviving more than 1 year was 12.3 per cent. The most worrisome late complications are neurological toxicities as well as carotid rupture. Fortunately, these devastating complications occur rarely, even in patients who receive large lifetime radiation doses.

**Recommendations**

• **Chemo re-irradiation appears to improve loco regional control, and may have some benefit for overall survival, at the risk of considerable acute and late toxicity. Benefit must be weighed carefully against risks, and patients must be counselled appropriately (R)**
• **Target volumes should be kept tight and elective nodal irradiation should be avoided (R)**

### Treatment volume definition

In re-irradiation, the potential benefit and toxicity of elective nodal irradiation need to be carefully considered, since the risk of toxicity is generally related to the volume of tissue irradiated. The literature suggests that the major risk of recurrence is within the region of gross recurrent disease. The probability of isolated failure in the electively treated areas is low. Treatment volume of the gross tumour should be expanded by a safety margin of 1–1.5 cm. Prophylactic treatment of draining lymphatic regions is generally avoided. In areas closely abutting critical structures, the margin may be smaller to reduce the risk of complications. After surgical resection, only the tumour bed of the high-risk areas (e.g. positive margin and extracapsular extension) is usually targeted.

## Best supportive care

Palliative and best supportive care should be offered routinely as part of the management package of all recurrence patients, even in the case of those who are being treated curatively. The early involvement of the palliative care physician can help control symptoms in the lead up to curative or palliative treatment. Furthermore, it provides a more seamless transition into palliative care if required. Involvement of a palliative care physician gives the patients confidence that their symptoms will be managed regardless of the outcomes of the treatment, and also can speed up the provision of support for patient and family at home.

### Key points

• Recent evidence suggests that outcomes of the management of recurrence are not as dire as is widely considered
• Evaluation and careful selection of patients with recurrence is the crux of successful management
• PET CT scanning is the most effective imaging method for the evaluation of recurrence
• Surgery appears to give the best outcomes for the management of recurrence, especially if HPV positive, but also has a high complication rate
• Patients who have the best outcomes from treatment are those with small recurrences and second primaries who do not smoke or who have stopped smoking, and have good performance status, and in whom the tumour can be completely removed with no involved margins, especially if chemoradiotherapy can be given afterwards if indicated
• The standard regimen for first-line palliative chemotherapy is cisplatin, 5-FU and cetuximab. However some patients may not be able to tolerate it
• Re-irradiation using tight target volumes may improve locoregional control, but does carry significant risk of toxicity
• Patients with recurrence often have significant symptoms, and should be offered best supportive care interventions regardless of the intent of therapy, as they can benefit from assessment and management by pain control teams and other clinicians.
